# Fluorescence cholangiography during laparoscopic cholecystectomy in a patient with situs inversus totalis: a case report and literature review

**DOI:** 10.1186/s12893-017-0242-x

**Published:** 2017-04-20

**Authors:** Narongsak Rungsakulkij, Pongsatorn Tangtawee

**Affiliations:** Department of Surgery, Faculty of Medicine, Ramathibodi Hospital, Mahidol University, Bangkok, 10400 Thailand

**Keywords:** Situs inversus, Situs inversus totalis, Laparoscopic cholecystectomy, Fluorescence cholangiography, Cholelithiasis

## Abstract

**Background:**

Situs inversus totalis is a rare autosomal disorder in which the patient’s affected visceral organs are a perfect mirror image of their normal positions. Surgery in these patients is technically challenging. Minimally invasive surgery such as laparoscopic cholecystectomy is the standard treatment for symptomatic cholelithiasis, but it can be difficult to perform. Laparoscopic cholecystectomy in patients with situs inversus totalis may be even more technically challenging. Fluorescence cholangiography is a new innovation in the field of navigation surgery. This procedure is safe and easy to perform, its findings are easy to interpret, and it does not require a learning curve or radiographs. It can be used in real time during surgery to identify extrahepatic biliary structures.

**Case presentation:**

We herein report a case of situs inversus totalis in a Thai patient with a history of biliary pancreatitis. He underwent laparoscopic cholecystectomy with intraoperative fluorescence cholangiography. The operation was successfully completed without complications. To the best of our knowledge, this is the first case report of the use of fluorescence cholangiography during laparoscopic cholecystectomy in a patient with situs inversus.

**Conclusion:**

Fluorescence cholangiographyis a new navigational surgical technique with which to identify extrahepatic biliary structures. It can be used as an adjunct technique during laparoscopic cholecystectomy to avoid biliary tract injury in difficult cases.

**Electronic supplementary material:**

The online version of this article (doi:10.1186/s12893-017-0242-x) contains supplementary material, which is available to authorized users.

## Background

Situs inversus totalis (SIT) is a rare autosomal recessive disorder with an incidence of 1 in 5000 to 20,000 live births. The anatomy of patients with SIT is a perfect mirror image of the normal positions of their visceral organs [1].

Surgery in patients with SIT is technically challenging. Minimally invasive surgery such as laparoscopic cholecystectomy (LC) is the standard treatment for symptomatic cholelithiasis and may be difficult to perform. However, LC in patients with SIT may be even more technically challenging [2–6]. Although many previous case reports have described the performance of LC in patients with situs inversus no standard technique has been established for these patients.

Optical or real-time surgery is being increasingly reported in the literature [7, 8]. One technique used in optical surgery is fluorescence cholangiography (FC) [9]. This method involves the administration of indocyanine green (ICG) by intravenous injection 30 min before surgery. ICG is taken up by the liver then excreted exclusively in the bile. The excitation of protein-bound ICG by near-infrared light causes it to fluoresce, thereby delineating components of the biliary system for the surgeon. Fluorescence and imaging is achieved through a system comprising a small control unit, a charge-coupled device camera, a xenon light source, and a 10-mm laparoscope containing specially coated lenses that transmit near-infrared light. FC is a feasible, low-cost, expeditious, useful, and effective imaging modality. Additionally, it is safe and easy to perform, its findings are easy to interpret, and it does not require a learning curve or radiographs. FC can be used during surgery to identify extrahepatic biliary structures [10, 11].

We herein report a case of cholelithiasis in a Thai patient with SIT who presented with a history of biliary pancreatitis. FC was performed during LC. To the best of our knowledge, this is the first such case reported in the literature.

## Case presentation

A 32-year-old man was referred to our hospital for further treatment. He had a known history of SIT. He had been admitted to the previous hospital with acute abdominal pain, and biliary pancreatitis was diagnosed. Ultrasonography showed multiple small gallstones. He was treated conservatively until clinical improvement was noted. After discharge, he was referred to our hospital. In our outpatient clinic, he was well with no fever, abdominal pain, or jaundice.

## Investigation

**Fig. 1:**
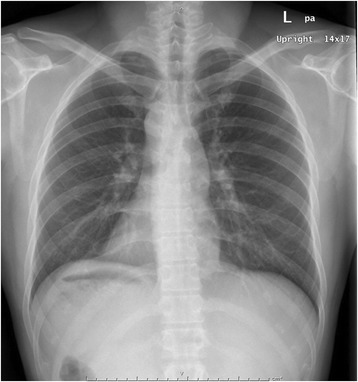
Chest radiograph findings. Dextrocardia is evident

**Fig. 2:**
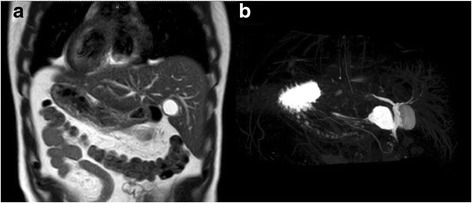
**a** Coronal T2W show mirror image of visceral organs. **b** Magnetic resonance cholangiopancreatography show the gallbladder is present on the left side of the abdominal cavity

The results of laboratory investigations, including liver function tests, were normal. A chest radiograph showed dextrocardia, confirming the presence of situs inversus (Fig. 1). He then underwent magnetic resonance cholangiopancreatography for evaluation of the common bile duct. Although no common bile duct stones were found, a mirror image of the intra-abdominal organs was evident, confirming SIT (Fig. 2). The gallbladder was situated on the left side. The patient was scheduled for elective LC.

## Management

**Fig. 3:**
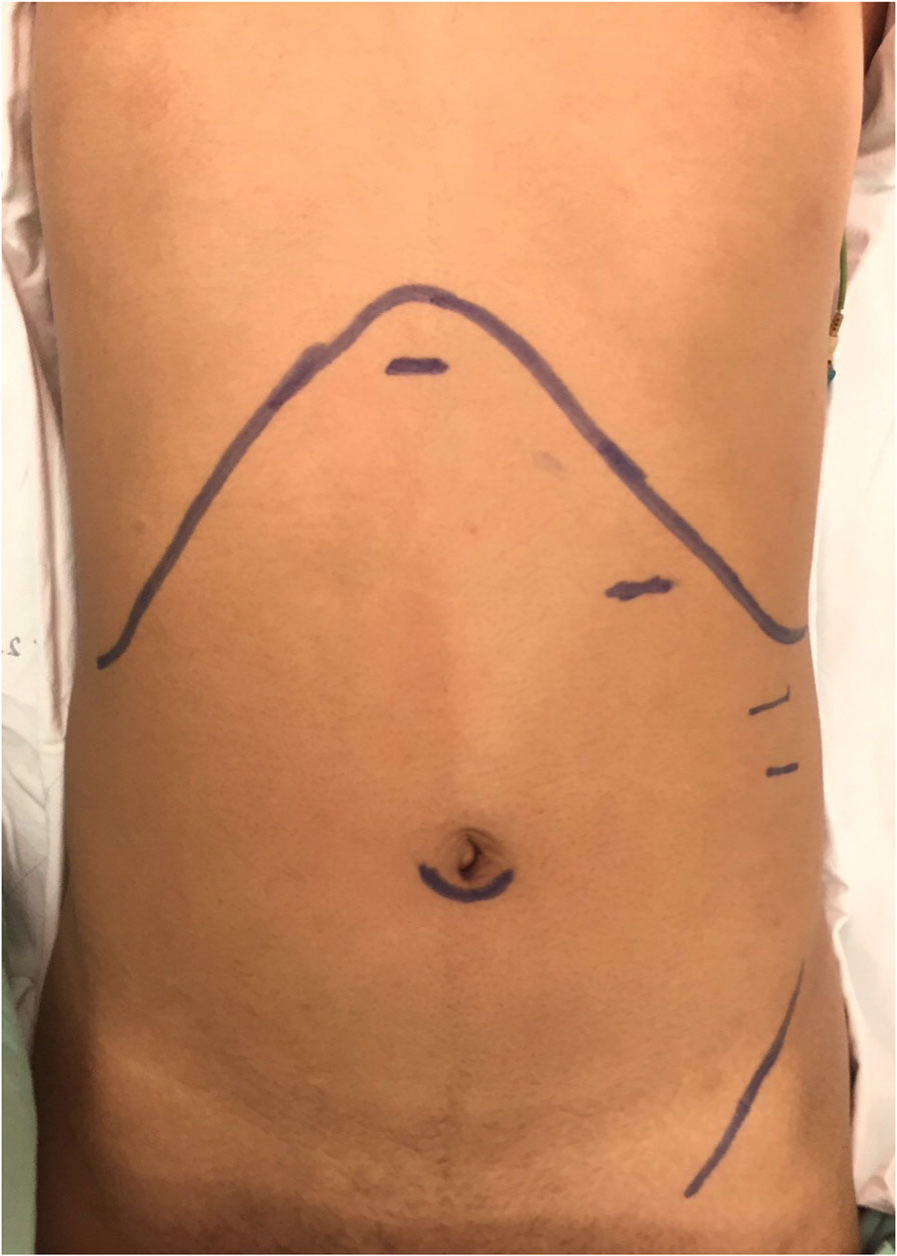
Locations of port insertion



During the operation, the patient was placed in the supine position near the right edge of the operative bed instead of the usual left side. The surgeon stood at the right side of the patient with the camera assistant, and the first assistant stood on the left side. The surgical incision was performed as shown in Fig. 3. A 12-mm subumbilical trocar was inserted by the open technique, and pneumoperitoneum was then established with carbon dioxide gas. A 0° laparoscope was inserted as shown in the video and three additional ports were inserted under direct vision. The first assistant then grasped the fundus of the gallbladder and retracted it cranially. Before dissection, we performed FC to identify the biliary tree as shown in the video fluorescent light was observed in the gallbladder and common bile duct. The infundibulum was then retracted laterally by the surgeon’s left hand. Dissection was performed to identify Calot’s triangle, and FC was repeated. We were able to identify the common bile duct, cystic duct, and cystic artery. No abnormal fluorescent light was present in the area between the cystic duct and liver edge. The cystic duct and artery were then individually clipped and safely divided, and the gallbladder was dissected from the liver bed by electrocautery. Hemostasis was performed, and the gallbladder was removed in a retrieval bag through the camera port. The operative time was 45 min. The patient recovered uneventfully and was discharged from hospital the day after the operation.

**Additional file 1:** Laparoscopic cholecystectomy in situs inversus totalis patient with using fluorescence cholangiography during the operation.


## Discussion

LC is the standard treatment for symptomatic cholelithiasis. In patients with SIT, however, LC is often technically demanding. Because of the unusual orientation, especially for right-handed surgeons, bile duct injury may easily occur [12]. Many previous reports have described LC in patients with SIT, but no standard techniques have been established for these patients [2–6].

Since Campos and Sipes [13] first reported LC in a patient with SIT in 1991, more than 50 cases of LC in patients with SIT have been reported without complications, even in patients with acute cholecystitis [2–4, 14, 15]. Thus, LC is not contraindicated in patients with SIT. However, such patients are at higher risk of complications because the mirror image of the anatomy is an unusual orientation [16]. Most previous reports have concluded that LC in patients with SIT is technically challenging and requires surgeons who are experienced in laparoscopic procedures and hepatobiliary surgery [3–6].

The most common surgical technique used by right-handed surgeons is the four-port technique with an incision created as a mirror image of the conventional incision [6, 17, 18]. The surgeon stands on the right side of the patient, the first assistant stands on the left side, and the camera assistant stands on the right side next to the surgeon. Left-handed instruments are used to grasp Hartmann’s pouch, and the right hand is used in the mid-clavicular port for dissection [3, 18].

FC is a recent innovation in optical surgery. ICG is intravenously injected about 15 to 30 min before the operation, and a special laparoscope containing specially coated lenses that transmit near-infrared light is used. FC is a feasible, low-cost, expeditious, useful, and effective imaging modality. It is safe and easy to perform, and its results are easy to interpret. Many previous reports have described the use of FC during LC to visualize the extrahepatic biliary anatomy and help to avoid bile duct injury [9–11]. This technique has been proven feasible, repeatable, and inexpensive [11]. The critical point in performing LC in patients with SIT is similar to that in patients without SIT; namely, that the critical view of safety must be identified [16, 19, 20]. In the present case, FC was used as a guidance technique with which to identify the biliary tree during the operation, especially at Calot’s triangle. This allowed for identification of the critical view of safety, making the operation less difficult. In such cases of difficult LC, as in patients with SIT, FC would be very helpful for surgeons to delineate the extrahepatic biliary tract. Even for less experienced surgeons, the use of FC guidance during careful dissection of Calot’s triangle could help to avoid biliary injury.

## Conclusion

LC in patients with SIT is a technically challenging procedure that requires an experienced surgeon. FC is a new navigational surgery technique that can be used to examine the extrahepatic biliary tree and serve as an adjunct during LC in difficult cases, helping to avoid biliary tract injury.

## Abbreviations

FC: Fluorescence cholangiography
ICG: Indocyanine green
LC: Laparoscopic cholecystectomy
SIT: Situs inversus totalis
